# “Treatment is both psychological and medical”: applying mixed methods to the understanding of mental and behavioral health in pediatric specialty medicine

**DOI:** 10.3389/fped.2025.1516773

**Published:** 2025-11-24

**Authors:** Desireé N. Williford, Jennifer Kelleher, Danielle M. Davidov, Christina L. Duncan

**Affiliations:** 1 Division of Behavioral Medicine and Clinical Psychology, Cincinnati Children’s Hospital Medical Center, Cincinnati, OH, United States; 2 Department of Psychology, West Virginia University, Morgantown, WV, United States; 3 School of Public Health, West Virginia University, Morgantown, WV, United States

**Keywords:** pediatric psychology, psychosocial, healthcare, subspecialty care, multidisciplinary, interdisciplinary

## Abstract

**Introduction:**

Mental and behavioral health (MBH) access varies in pediatric specialty medicine and rural healthcare. This study aimed to characterize healthcare professionals' perceptions and experience with MBH services and readiness for change.

**Methods:**

Thirty rural healthcare professionals completed validated questionnaires and semistructured interviews designed to meet study objectives. A convergent mixed methods design with a comparison method of interpretation was followed.

**Results:**

Thematic analysis yielded three major themes highlighting the prevalence of MBH concerns in practice, global perceptions of MBH care, and related barriers/facilitators to implementation. Current levels of MBH integration varied though overall readiness for change was high.

**Discussion:**

Findings highlight stakeholder perceptions about the role of MBH in pediatric specialty medicine. Key considerations for those who utilize or plan to adopt integrated MBH care at various levels are discussed, including readiness to change and other structural (e.g., organizational) or individual factors serving as facilitators and barriers to implementation.

## Introduction

Pediatric mental health is a public health crisis and a leading cause of global disease burden (1, 2). Typical onset of mental health conditions is prior to 18 years of age and symptoms often persist into adulthood (1, 3). One in eight pediatric patients have a mental or behavioral health (MBH) condition warranting intervention (i.e., impairing development of well-being); yet many are not receiving care (1). The COVID-19 pandemic exacerbated MBH concerns globally (4, 5); however, pre-pandemic, MBH concerns and reduced health-related quality of life were well-documented for youth with complex medical needs (6). As risk and symptoms are likely to persist over time, youth with chronic health conditions warrant particular attention (7).

Areas of pediatric specialty medicine have recognized the need for MBH care standards, including routinely screening/assessing, preventing, and intervening upon MBH symptoms (8–11). For example, US News and World Report rankings are dependent upon access and integration of MBH services, including the implementation of MBH screening. Pediatric primary care has also established frameworks detailing strategies for how medical teams can integrate MBH specialists into standard care (12). *A Standard Framework for Levels of Integrated Healthcare* (13), for example, illustrates MBH integration as a continuum, ranging from minimal coordination (coordinated care) to full collaboration (integrated care) where roles and operations of medical and MBH care are part of a single, merged practice.

Despite literature supporting the efficacy of various care models, psychosocial screening tools, and MBH interventions, few studies have examined specific barriers and facilitators in these settings or ways to enhance implementation of MBH into pediatric specialty care and even fewer studies have examined these factors in the context of rural healthcare systems. A contributing factor to this void is the lack of partnership between healthcare professionals and administrators in developing the models they are expected to implement and/or utilize. For example, recent reports and commentaries describe how growth in healthcare administration has outpaced growth of physicians. This has facilitated a gap such that physicians and the teams they manage may feel burdened by increased scrutiny, workload demands, and regulatory requirements, contributing to physician burnout and fatigue (14, 15). This perpetuates a science-to-practice gap and misalignment between research, administrative regulations and practice operations, and the lived experiences of barriers and facilitators of healthcare professionals providing care to patients. Further, other recent research has discussed barriers experienced by healthcare professionals, including physicians, with the integration of psychosocial and MBH screening. A recent qualitative study in oncology, for example, described despite expectations to implement MBH screening, those expected to implement often perceived limited institutional support and commitment and often felt burdened by these practices without sufficient resources to implement or act upon screening results (16).

In academic medical settings, including smaller, under resourced, and rural healthcare systems, healthcare professionals such as physicians hold clinical care appointments in addition to clinic/division and hospital administrative roles. This provides a unique opportunity to evaluate preferences, facilitators, barriers, and other key considerations related to acceptability and feasibility of MBH integration in pediatric specialty care. The current study thus brings various healthcare professional and administrator perspectives together for this purpose.

### Aims and objective

Using a convergent mixed methods (QUAL-quant) design, the study aimed to (1) identify key themes from semi-structured interviews about perceptions of MBH care and applicability to current practice; (2) examine levels of current MBH coordination/integration and readiness for change domains and (3) integrate findings to identify and explore relations between qualitative and quantitative indicators of lived experiences with MBH in everyday practice. While aims were exploratory, it was anticipated that (1) thematic analysis of semi-structured interviews would result in the identification of varied perceptions and experiences with MBH services within and across clinical care teams (i.e., pediatric specialty areas) and (2) that relations could be drawn between qualitative thematic data, current levels of MBH coordination/integration among specialty areas, and quantitatively-collected readiness for change factors.

## Methods

### Participants

Thirty-two healthcare professionals in pediatric specialty care were recruited from a hospital in the Appalachian region of the U.S. Per specialty area represented in our data, we attempted to recruit one healthcare professional with hospital or department-level administrative responsibilities plus at least two team members. Inclusion criteria for all participants were as follows: (1) healthcare professionals (e.g., physicians, nurse practitioners, registered nurses, dieticians, social workers, psychologists) who practice in a pediatric medical specialty (i.e., provide direct patient care or clinical teaching/supervision of individuals providing direct patient care, has direct interactions with other healthcare professionals and patients/families as part of scope of practice); (2) at least 1 year of practice/experience in specialty area; and (3) employed by the recruitment site for at least 6 months. Administrator-specific criteria also included intimate knowledge/experience with specialty practice operations as determined by 1) formal administrative designation or title (e.g., Director, Chief, Chair, Vice Chair) and (2) leadership role(s) and responsibilities within the specialty area or hospital (i.e., leads faculty and staff; involved in the development and/or implementation of hospital/specialty area regulatory procedures and policies; facilitates research, educational, clinical services, strategic planning, development, resource management). Students/trainees were excluded.

Participants were 30–62 years of age, with an average age of 42.5 years (*SD* *=* 9.9) for non-administrative health professionals and 50.7 years (*SD* = 9.1) for administrators. Most participants had 5 or more years of experience in their specialty area (*n* = 20, 86.9% of non-administrative healthcare professionals; *n* = 7, 100.0% of administrators). Most non-administrative healthcare professionals were female (*n* = 19, 87.0%) and White (*n* = 22, 95.7%). Most had an advanced degree (*n* = 17; 73.9%) and were trained in nursing (*n* = 12, 52.2%), medicine (*n* = 4, 17.4%), or social work (*n* = 4, 17.4%). Most administrators were also female (*n* = 4, 57.1%) and White (*n* = 5, 71.4%). All (*n* = 7, 100%) held an advanced (doctoral) degree in medicine. Additional details, including roles held on the clinical team, are described in Table 1.

**Table 1:** Sample characteristics.

Participant Demographics		Non-administrative healthcare professionals (*N* = 23)		Administrators (*N* = 7)	
		Mean/*N*	SD/Percent	Mean/*N*	SD/Percent
Age (in years)		42.5	9.9	50.7	9.1
Gender	Female	19	87.0%	4	57.1%
	Male	4	17.4%	3	42.9%
Race^a^	White	22	95.7%	5	71.4%
	Black or African American	0	0.0%	1	14.3%
	Asian	1	4.3%	0	0.0%
	Multiracial	0	0.0%	1	14.3%
Highest Level of Education	Bachelor's Degree	6	26.1%	0	0.0%
	Master's Degree	12	52.2%	0	0.0%
	Doctoral Degree^b^	5	21.7%	7	100.0%
Highest Degree Earned/Healthcare Professional Type	Medicine (Physician)	4	17.4%	7	100.0%
	Nursing (Nurse, Nurse Practitioner)	12	52.2%	0	0.0%
	Psychology (Psychologist)	2	8.7%	0	0.0%
	Social Work (Social Worker)	4	17.4%	0	0.0%
	Nutrition/Dietetic (Dietician)	1	4.3%	0	0.0%
Roles^c^	Direct Patient Care	22	95.7%	7	100.0%
	Clinical Teaching	15	65.2%	6	85.7%
	Research	7	17.4%	6	85.7%
	Administrative Assignment	0	0.0%	7	100.0%
	Leadership Roles^d^	3	13.0%	7	100.0%
	Other	2	8.7%	7	100.0%
Years of experience in specialty area	<5 years	3	13.0%	0	0.0%
	5–10 years	10	43.5%	1	14.3%
	>10 years	10	43.5%	6	85.7%

a Racial/Ethnic categories included: White, Black or African American, Asian, Hawaiian or Pacific Islander, American Indian or Alaskan Native, Hispanic or Latino, Multiracial, and Other. All not listed above were not represented in the current sample.

b For all administrators, doctoral degree refers to medical degree (physician).

c Most participants indicated their positions involved multiple roles (non-administrative healthcare professionals: *n* = 15, 65.2%; administrators: *n* = 7, 100%). As such, the total *n* for roles is greater than the number of participants.

d These roles did not qualify these individuals as administrators for their specialty area.

### Procedure

Procedures approved by an Institutional Review Board (Protocol #1809263858). Reporting on this mixed-methods study follows the Journal Article Reporting Standards for Qualitative Research in Psychology, specifically the Mixed Methods Article Reporting Standards (17).

#### Recruitment and engagement

Participants were recruited across specialties and training backgrounds to capture breadth and depth in perspectives. Given potential barriers to recruiting and retaining healthcare professionals in research (e.g., time, fear of evaluation), evidence-based recruitment and engagement strategies (i.e., email, recruitment letters/flyers, in-person meetings, physician champion) were implemented (18, 19). Two authors (DNW and JK) conducted recruitment and engagement procedures and served as qualitative interviewers for the study.

While 32 healthcare professionals were enrolled, data from 30 participants were available for analysis. One healthcare professional was withdrawn due to lack of response from other healthcare professionals in their specialty area. Another participant withdrew due to an employment-related research restriction. Of the 11 pediatric specialty teams contacted, nine participated: Gastroenterology/Hepatology/Nutrition, Cystic Fibrosis, Neurology, Cardiology; Endocrinology; Hematology/Oncology, Nephrology/Hypertension; and the Pediatric Infusion Center. Psychology/Behavioral Medicine was also represented as a team and included MBH clinicians who deliver these services within one or more of the enrolled medical specialties. Of note, the Pediatric Infusion Center serves patients from multiple medical teams, including several represented in the study. Nevertheless, services provided in the Infusion Center were generally independent from those in the specialty clinic settings leading to its denotation as a separate specialty team. An exception was Hematology/Oncology given significant collaboration/coordination of care. Thus, comments made by Infusion Center participants specific to Hematology/Oncology services (and clarified as such during the interview) were described as Hematology/Oncology team findings. To maintain confidentiality due to the small sample sizes of each team (*n* ≤ 4 each), all team-specific data reported henceforth will use a randomly assigned team number (Teams 1–9). Among teams represented in the study, enrollment ranged from 66.7% to 100.0% and refusal ranged from 0.0% to 17.0%. According to sampling procedures, not all members of a team were approached; thus, enrollment and refusal rates were calculated based on the total number of individuals approached per team.

#### Sampling techniques

Consistent with the convergent mixed methods design, qualitative and quantitative data collection and analysis occurred concurrently (20). Purposive and theoretical sampling fostered representation of diverse professional backgrounds, roles, and experiences within/across settings and strategic recruitment of individuals/specialty areas with varied MBH experiences and care models. The first author (DNW) maintained a log to track recruitment decisions and use of these sampling strategies. For example, during a few healthcare professional interviews, it was recommended that the research team talk to an individual who holds a specific role within their team to more comprehensively answer some of our questions. This often led to our team attempting to recruit these individuals to ensure inclusiveness as these perspectives could influence resulting themes. Analytically, these approaches informed the determination of codebook stability, or the inability to identify new codes or themes not categorized within the existing codebook (21–24).

### Measures

#### Qualitative semi-structured interview guide

Participants completed a semi-structured interview (60 minutes, sometimes split into two sessions). Interviews were conducted by phone or in-person with a trained interviewer (DNW or JK) and were audio-recorded and transcribed. The interview guide was developed in consultation with an expert in integrated primary care and piloted with ineligible healthcare professionals. Participants were compensated $50 (gift card).

#### Quantitative measures

Participants completed electronic study questionnaires via the Research Electronic Data Capture (REDCap) platform (25).

##### Healthcare professional information form (PIF)

The PIF is a study-specific measure of participant demographics, work environment, and role(s) within specialty teams.

##### Readiness for change questionnaire (RCQ)

To assess readiness for organizational change, participants completed the 25-item RCQ which has been previously validated with healthcare professionals (26, 27). Items were rated on a 7-point, Likert-type scale (“Strongly Disagree” to “Strongly Agree”) and correspond to four readiness for change domains (*Appropriateness*, *Management Support*, *Change Efficacy,* and *Personally Beneficial*) conceptually consistent with Holt and colleagues' (2010) framework. With permission, RCQ language was slightly modified (e.g., “management” changed to “director/administrator,” “organization” changed to “healthcare setting”). The RCQ demonstrated acceptable reliability across subscales (*α* = .77-.88).

##### Integrated practice assessment tool, version 2.0 (IPAT)

At their scheduled interview, administrators were administered the 8-item IPAT to determine their team's current level of MBH coordination/integration (28). Employing a decision-tree model and series of yes/no questions, the IPAT categorized each specialty medical team into either: *Coordinated Care* (Level 1, Minimal Collaboration and Level 2, Basic Collaboration at a Distance); *Co-Located Care* (Level 3, Basic Collaboration Onsite and Level 4, Close Collaboration Onsite with Some Systems Integration); or *Integrated Care* (Level 5, Close Collaboration Approaching an Integrated Practice and Level 6, Full Collaboration in a Transformed/Merged Integrated Practice.) For two specialty teams, an administrator was not required to complete the IPAT per eligibility criteria and team structure. IPAT responses for the remaining seven teams were obtained and audio-recorded for confirmation of scoring accuracy.

Though intentions were to calculate one IPAT score per team, four teams had unique circumstances warranting calculation of a second IPAT score. A secondary IPAT score was calculated when the specialty team had (1) a recent change in their level of MBH support (i.e., loss of contract/funding) or (2) differing levels of MBH support within their specialty (i.e., one clinic with a higher level of MBH integration than most clinics). These circumstances resulted in pre- and post-change IPAT scores or an overall and clinic specific IPAT score, respectively.

Both scores were reported descriptively; however, the score reflective of most clinics/care provided within the specialty area or current (post-change) IPAT levels was used in between-group analyses.

### Sample size and data analysis

#### Qualitative and quantitative analyses were conducted using NVivo 12 plus and IBM

SPSS Statistics (Version 26), respectively. Sample size was determined by extant literature (23, 29). Qualitative thematic analysis was led by the first author (DNW), with significant involvement of the second author (JK), both of whom served as interviewers for the current study. Additional coders (*n* = 2) included trained undergraduate research assistants not involved with study procedures. Each transcript was reviewed by 3 coders, with at least one coder not involved in study conduct. Remaining authors (DMD and CLD) held supervisory/mentorship roles in study conduct and data analysis. Given inherent differences in sample sizes across groups (i.e., fewer administrators as compared to healthcare professionals), group differences were quantitatively assessed using nonparametric statistics, specifically using Mann–Whitney, Kruskal–Wallis tests. There were no missing data.

##### Aim 1

The first aim examined perceptions regarding the role and feasibility of implementing MBH care in pediatric specialty care settings. The four trained coders followed an iterative thematic analytic process involving five stages (30–34). First, coders became familiarized with the data via independent review without *a priori* hypotheses. Each coder then compiled an initial list of codes, or patterns of conversation, among interviews. During the second stage, coders collectively discussed independently generated codes, allowing for codes to be grouped together, refined, and solidified into a condensed number of codes. This process employed both deductive and inductive coding methods and incorporated reliability checks to create a finalized codebook (Stage 3). Specifically, some codes pulled from the data were influenced by questions included in the semi-structured interview (deductive) and others emerged as new, related topics were brought by participants and observed during independent reviews and team discussions (an inductive process). The fourth stage involved a second independent review using the codebook, with the addition of a new coder not involved with the first round of review for reliability purposes. A similar discussion post-review occurred as well as calculation of inter-rater reliability during the fifth and sixth levels of analysis. These stages allowed for further refinement of the codebook, resolving discrepancy to 100 percent consensus, and establishing a finalized coding frame including thematic clusters or categories of codes and intersections/relations among codes (i.e., creation of sub-themes).

##### Aim 2

The second aim identified current levels of readiness to change and levels of coordination/integration across medical teams. Descriptive statistics were calculated for the RCQ (*Appropriateness, Management Support, Change Efficacy*, and *Personally Beneficial* scales) and IPAT by healthcare professional type (administrator vs. non-administrative healthcare professional). A secondary goal was to characterize and explore group differences in readiness for change factors. Mann–Whitney and Kruskal–Wallis nonparametric tests were conducted to examine potential differences in RCQ scores by demographic factors and the level of coordination/integration for each specialty area.

##### Aim 3

The third aim integrated qualitative (Aim 1) and quantitative (Aim 2) findings according to a convergent design and integration through connecting and merging approaches (35). As the present study involved quantitative measures and qualitative interviews were conducted concurrently, connecting occurred through sampling (i.e., participants completed both procedures) and the convergent study design. This process involved comparing two sets of data to draw a more complete conceptual picture, determine how results inform or expand upon each other, describe different aspects of the research question, and visually integrate data utilizing a mixed methods matrix (20, 36).

## Results

### Qualitative findings

Thematic analysis of interview data revealed three overarching themes, each with corresponding sub-themes (see Table 2).

**Table 2:** Thematic analytic findings.

Emergent themes and subthemes	Definition
Theme 1: Prevalence of MBH Concerns & Relevance to Pediatric Specialty Medicine a) Overlap between psychosocial concerns and physical health, medical complexities, and/or medical treatment b) Specific diagnoses or MBH concerns c) Current or prospective roles of MBH clinicians d) Other psychosocial concerns impacting medical care or patient functioning e) MBH referrals and related processes f) Guidelines/policies around mental and behavioral health	References to psychosocial and MBH concerns common in the specialty area's population, the types of MBH professionals (clinicians) involved with the clinic, and the roles/services these clinicians offer. Guidelines/policies around MBH were included.
Theme 2: Perceptions of Coordinated/Integrated MBH Care a) Perceived need for increased MBH services b) Thoughts around models of coordination/integration care and any evidence of services meeting the definition of coordinated care, co-located care, or integrated care	Discussions around the need for MBH services as well as beliefs around facets of the coordination/integration care model. This included perceptions around the current care model for each specialty area, the ideal/preferred model, which model(s) were viewed as most feasible, and any discussion around team dynamics that provided evidence of a care model.
Theme 3: Perceived Feasibility and Acceptability of Coordinated/Integrated MBH Care a) Organizational-level considerations b) Healthcare Professional-level considerations c) Patient population-level considerations	References to perceived barriers and facilitators to MBH services across levels (i.e., healthcare organization and system, Healthcare Professional, patient/family).

#### Theme 1: prevalence of MBH concerns and relevance to pediatric specialty medicine

This theme broadly captured references to the prevalence and relevance of MBH concerns to routine subspecialty care. Six sub-themes were identified.

##### Overlap between psychosocial concerns and physical health, medical complexities, and/or medical treatment

This subtheme included statements about the frequency and relevance of psychosocial concerns to the care of patients and families. Healthcare professionals also discussed that psychosocial concerns are not always related to medical concerns but could exacerbate or contribute to chronic illness management.

**Healthcare Professional (Team 4):** “[MBH concerns] may or may not actually be related to what they have going on medically…the treatment is both psychological and medical and you have to do both…and if you don’t do both parts, they don’t get better.”

##### Specific diagnoses or MBH concerns

This subtheme included references to common MBH concerns observed in practice, including anxiety; depression/suicidal ideation, externalizing and risk-taking behaviors; attention-deficit/hyperactivity disorder; and autism spectrum disorder and other developmental or intellectual disabilities. Healthcare professionals also noted differential risk or prevalence of MBH challenges among specific subgroups within their populations (e.g., based on developmental, chronicity, and/or disease severity considerations).

**Administrator (Team 5):** “…Anxiety, depression, infantilization…there’s suicidal ideation…a lot of fear…there’s so many bad habits, bad behavioral responses to things that happen to kids…[and] parents in the hospital..they just result from all these horrible things that people aren’t coping with or are talking about or aren’t working through….”

##### Current or prospective roles of MBH clinicians

This subtheme captured references to current or prospective roles and support provided by MBH clinicians to medical teams. Examples include providing referrals or resources; clinical intervention and/or assessment; and serving a consultative role to patients, families, and staff. Healthcare professionals and administrators also discussed “gaps” MBH clinicians fill in routine practice and how psychosocial concerns were/were not addressed without MBH support.

**Healthcare Professional (Team 4):** “[The MBH clinician] is usually asked to help with kids who are having psychological contributors that are impacting their medical problems or…that the team has gotten a sense of and feels like it’s not being addressed.”

##### Other psychosocial concerns impacting medical care or patient functioning

This subtheme referenced other non-diagnostic psychosocial concerns and patient/familial considerations raised by participants (e.g., disease burnout, treatment engagement, treatment responsibility, adherence, and self-management behaviors). These concerns were described as impacting prognosis, treatment effectiveness, or overall patient functioning.

**Healthcare Professional (Team 3):** “[Our MBH clinician is helpful] with…trying to come up with ways to encourage excitement with trying new things and problem solving [around barriers].”

##### MBH referrals and related processes

This subtheme captured references to internal or external MBH referral processes and follow-up, if applicable. Participants with limited MBH support noted barriers to follow-up on the referrals provided to patients and families. In these instances, families navigate the referral process more independently, unless members of the team are specifically knowledgeable about resources available. Further, healthcare professionals also discussed limited resources available in rural Appalachia, even when interested in providing referrals.

**Administrator (Team 5):** “[I refer more] older kids…because I can find them places. Younger kids, I can’t find them anywhere to go…it’s not for lack of [trying], there’s nowhere they can go, so there’s nowhere to send them..”

##### Guidelines/policies around MBH

This subtheme emphasized references to any guidelines or policies around MBH for the area of pediatric specialty. In most instances, healthcare professionals were unaware of any specific guidelines or policies and instead described variation in how/if MBH concerns were addressed by healthcare professionals within the team. For healthcare professionals aware of recommendations, the guidelines were often described as either vague or not feasible due to adoption/implementation barriers. Specific barriers noted were included within the “Perceived Feasibility and Acceptability of Coordination/Integration MBH Care” theme.

**Administrator (Team 8):** “…The [organization name omitted] actually lays out some [recommendations] for disorders… [but] they leave the mental health component fairly vague…it’s usually [the same general] recommendations, pretty much for everyone…A lot of it comes [from research]…but there’s not anyone in the field who has come out and said, ‘This is the exact model that [MBH care] should be provided in’.”

#### Theme 2: perceptions of coordination/integration MBH care services and models

This theme, incorporating two subthemes, captured participant preferences around coordination/integration MBH services and models.

##### Perceived need for increased MBH support

Participants frequently reported interest in coordination/integration MBH due to patients and family needs. Healthcare professionals described MBH clinicians as having nuanced skillsets and training helpful to both patients/families and the medical team, including allowing healthcare professionals to meet visit goals and objectives more efficiently. Healthcare professionals and administrators often described how their hospital system has historically undervalued/underemphasized MBH integration and the role services play in prevention of expensive, complex, and/or debilitating comorbidities.

**Administrator (Team 1):** “…[Patients] think they have come for medication and even if I asked [about MBH concerns], they would tell me something superficial. I think [the MBH clinician will] be better…she has the right tools to get that information from them.”

##### Thoughts around models of coordination/integration care and any evidence of services meeting the definition of coordinated care, co-located care, or integrated care

This subtheme highlighted discussion around the specific models of coordination/integration care according to the Standard Framework for Levels of Integrated Healthcare. Healthcare professionals discussed their preferences and perspectives around these models as well as articulated understanding and/or confusion about the terminology. They provided specific examples from within their practice and beyond (e.g., prior work experiences) with these varied models and highlighted general pros and cons of each approach.

**Administrator (Team 6):** “I would love to have a psychologist integrated right in our team. And he’s a person who sees every single patient in our clinic, maybe not every visit, but gets to know families and patients and…help them…[with] whatever they need. It’s a reliable, trusted, very qualified person who is not going to change…”

#### Theme 3: perceived feasibility and acceptability of coordination/integration MBH care

This theme highlighted perceived facilitators and barriers to the implementation of coordination/integration MBH Care at the organizational, professional, and patient levels.

##### Organizational considerations

This subtheme reflected thoughts, actions, behaviors, or reasons that discouraged the implementation of coordination/integration care models or increase of MBH services within their specialty area or organization more broadly. Barriers were discussed across the larger organization level as well as at the level of individual specialties and clinics. Examples include insurance and healthcare structure considerations; use of electronic medical records; healthcare growth/expansion; and the availability of staffing, time, space, and other resources.

**Healthcare Professional (Team 4):** “I think co-located care is really important…I'm a huge proponent of integrated care, but there are times where co-located makes the most sense, particularly when we are trying to ease way into changes within the system and/or when we do need psychological staff [MBH clinicians] to have more time and space than is appropriate for an integrated care setting.”

**Administrator (Team 9):** “It’s all about money,…having enough…[MBH clinicians] having enough physical space in the clinic, and finding a way to get those services reimbursed. Some of that is not sustainable, but….we have clinic patient rooms, exam rooms, and we have Healthcare Professional workstations. And the idea would be to have the [MBH clinician] have a workstation in the same physician work area…with the rest of the team and just available on an ad-hoc basis to say, ‘Hey, I just got done with this family. I think they could really benefit from some time with you.’ And then giving a warm hand-off.”

##### Healthcare professional-level considerations

Healthcare professionals and administrators also discussed barriers and facilitators at the individual Healthcare Professional level, including personal barriers around communication and interest or comfort with MBH topics. For example, healthcare professionals discussed difficulties in verifying follow-through with referrals provided, making coordinated care more difficult to navigate effectively and systematically. They also discussed concerns within the electronic medical record system, including stressors associated with “breaking the glass” to read MBH notes for coordination of care purposes.

**Administrator (Team 3):** “I don’t know how many people don’t end up going to their referrals and I think [obtaining] that feedback…could be improved…I personally don’t like breaking the glass [to enter a MBH note]….and some of the concerns obviously can’t be out there in the medical chart at large, I just don’t know how to better coordinate…”

**Healthcare Professional (Team 7):** “I would love to see a [MBH clinician] dedicated to our team…We can discuss the patient and see if there are any things that I can improve from the patient point of view, the [medical] issues. And in the meantime, I can transfer my thinking and my thought about the overall situation to the [MBH clinician] and we discuss what would be best for the patient. But definitely, I [would] encourage her or him, the [MBH clinician], to see the patient on his own. To take his time and to assess him as he feels like.”

##### Patient population-level considerations

Several perceived barriers for patients and families were mentioned by participants, such as patient disinterest (though described as rare), the large catchment area served by their hospital, patient population growth, and other demographic contributory factors, including social determinants of health, historical trauma (e.g., opioid crisis) and systemic barriers to healthcare heightened in rural, Appalachian, and other marginalized communities.

**Healthcare Professional (Team 4):** “I think coordinated care is challenging…First, being that we know that patients do not routinely follow through with recommendations from their medical [team] to go see a [MBH clinician]. So, if you see a patient, they say they have depression, you tell them that you need them to go to [practice name omitted] or another outpatient mental health facility, there’s about a 50% chance that they're actually going to go and follow up with that [for numerous reasons].”

**Administrator (Team 7):** “When someone calls me, finally admits [substance abuse] and says I need help…particularly as [their specialty care team] for so long, they think of us as their primary care doctor. So, I can’t say, ‘Yeah, that’s great you’re admitting it, go see your pediatrician or your whatever about it.’ Wouldn’t it be nice if I could’ve got [this patient] hooked at least on the phone with somebody who knew what to do, because I have no idea what to do with that.”

### Quantitative findings

#### Differences in RCQ scores

Non-parametric independent samples tests (i.e., MannWhitney, Kruskal–Wallis) assessed mean differences in RCQ scores based on demographics (Table 3). Significant differences were observed for the *Personally Beneficial* subscale, such that the scores of administrators (Mdn = 7.00) were higher than those of non-administrative healthcare professionals (Mdn = 6.33), U(*N*healthcare professionals = 23, Nadministrators = 7,) = 123.50, z = 2.21, *p* = .033. No significant differences were found for other subscales (i.e., *Appropriateness*, *Management Support*, *Change Efficacy*) based on healthcare professional type or other demographic variables (i.e., age, gender, years of experience, education level, area of specialty).

**Table 3:** Mean and Median Differences for RCQ Scores by Healthcare Professional Type.

RCQ subscale^a^	Mean score (SD)^b^	Median score (SD)^b^
Full Sample (*n* = 30)		
Appropriateness	6.31 (0.52)	6.3 (0.52)
Management Support	4.94 (1.14)	4.94 (1.14)
Change Efficacy	6.13 (0.62)	6.00 (0.62)
Personally beneficial	6.47 (0.58)	6.67 (0.58)
Non-Administrative Healthcare Professionals (*n* = 23)		
Appropriateness	6.21 (0.48)	6.3 (0.48)
Management Support	5.01 (0.93)	4.83 (0.93)
Change Efficacy	6.01 (0.59)	6.00 (0.59)
Personally beneficial	6.35 (0.61)^c^	6.33 (0.61)^c^
Administrators^d^ (*n* = 7)		
Appropriateness	6.63 (0.55)	7.00 (0.55)
Management Support	4.74 (1.74)	5.00 (1.74)
Change Efficacy	6.52 (0.57)	6.83 (0.57)
Personally beneficial	6.86 (0.26)^c^	7.00 (0.26)^c^

a No significant differences were found for the following scales based on healthcare professional type: *Appropriateness* [U(N_professionals_ = 23, N_administrators_ = 7,) = 118.50, z = 1.87, *p* = .061], *Management Support* [U(N_professionals_ = 23, N_administrators_ = 7,) = 75.50, z = -.25, *p* = .806], and *Change Efficacy* [U(Nprofessionals = 23, Nadministrators = 7,) = 114.50, z = 1.69, *p* = .091].

b Range = 1–7, with higher scores indicating greater readiness for change on a given domain.

c Significant at the *p* < .05 level.

d All administrators are physicians.

#### IPAT scores

Seven primary and four secondary IPAT scores were calculated. Among primary IPAT scores, minimal variation was observed, and all seven IPAT scores identified teams as *Co-Located Care* (Level 3, *n* = 2, 28.6%; Level 4, *n* = 5, 71.4%). Regarding within-specialty differences, secondary IPAT scores revealed increased variability, with higher levels of integrated care represented (*Co-Located Care:* Level 3, *n* = 2, 18.2% and Level 4, *n* = 6, 54.5%. *Integrated care*: Level 5, *n* = 2, 18.2% and Level 6, *n* = 1, 9.1%). No teams were categorized as *Coordinated Care* (Levels 1 or 2). Results are incorporated into the mixed-methods matrix described below. There were no significant differences found between IPAT and RCQ scores.

### Integration of qualitative and quantitative findings

**Figure 1:**
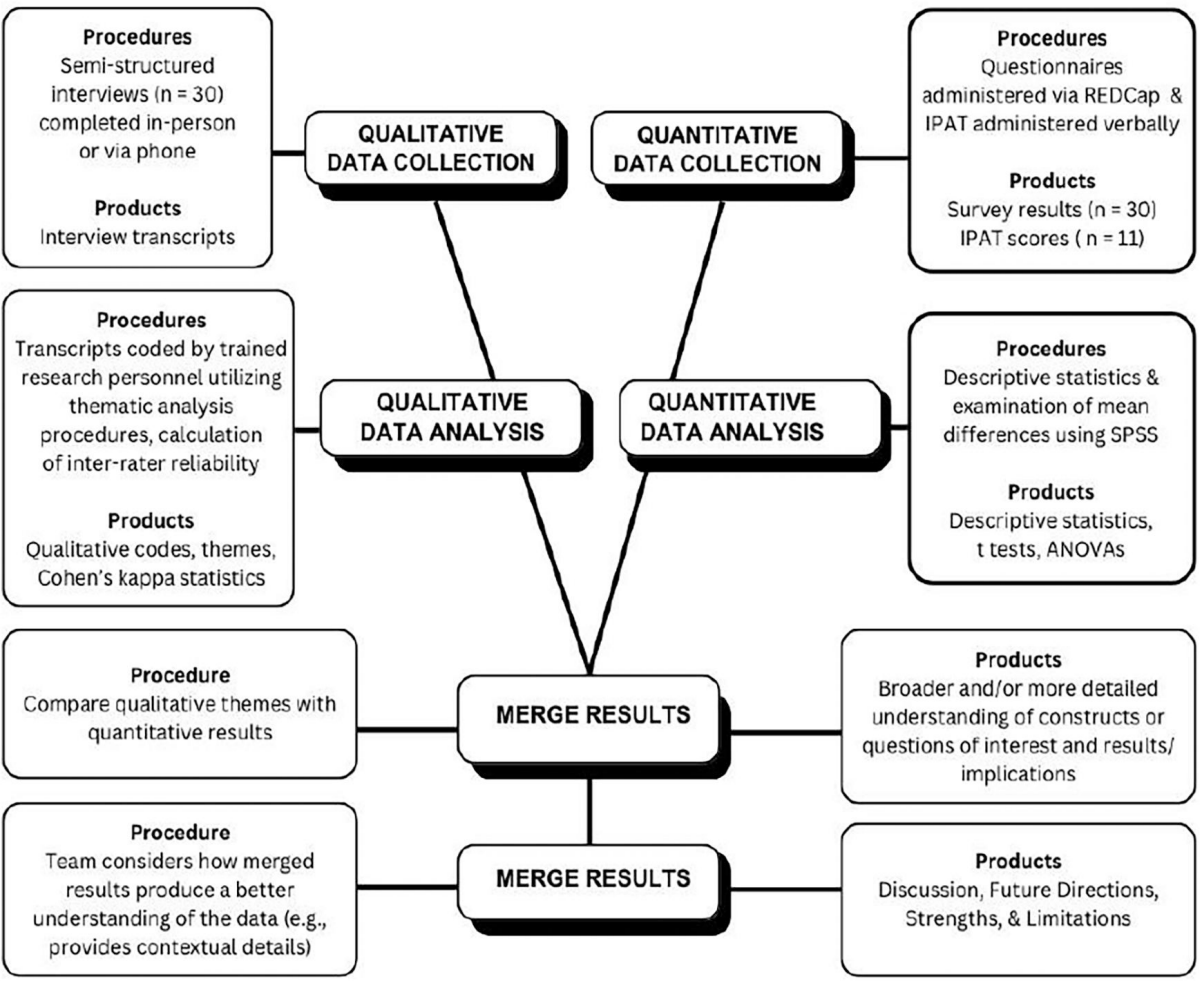
Convergent mixed methods design.

Integration of qualitative and quantitative findings followed a convergent mixed methods design using a comparison method of analysis (Figure 1). Comparing the two sets of data helped elucidate how results informed or expanded upon each other and described different aspects of the research question. We examined overlap between IPAT scores and readiness to change factors (RCQ scores) within and across teams and in relation to identified themes, specifically to better understand context of individual and team familiarity and experience working with MBH providers and addressing MBH concerns in their work (Theme 1), perceptions of care models (Theme 2) and specific facilitators and barriers (Theme 3), which may impact perceptions of MBH services or readiness for change. Outputs from this integrative process are represented in a mixed methods matrix (Table 4).

**Table 4:** Mixed methods matrix.

Team	Mean (SD) RCQ scores^a^	Representative quote(s)^b^	IPAT score(s)
TEAM 1^c^ (*n* = 3)	Appropriateness: 6.20 (0.85) Management Support: 5.06 (1.69) Change Efficacy: 6.28 (0.63) Personally Beneficial: 6.65 (0.51)	“I think when [the MBH clinician] was there…there are some issues that she can bring up…about stuff going on at home. [Patients often] just think they have come for medication and even if I asked [about MBH concerns], they would tell me something superficial. I think [the MBH clinician is preferred]…she has the right tools to get that information from them.” – *Administrator* “…I mean this is ideal, not realistic…[to consistently] use a screening tool [i.e., depression screening] for our patients to find out…what's bothering them… and then [provide] referrals for help.” – *Healthcare Professional* “…If we had somebody where we could just pick up the phone and say, ‘Hey, are you busy? Could you come over and see this patient?’ I think that would be ideal. Even if they're not physically in clinic at the time, if we just were able to call, like we [do with our call social worker when a patient does not have insurance]…if we could do something like that with the psychology [service].” – *Healthcare Professional*	Level 4 Co-Located (a particular clinic within Team 1)
			Level 3^d^ Co-Located (all other clinics within Team 1)
TEAM 2^e^ (*n* = 3)	Appropriateness: 6.37 (0.55) Management Support: 4.72 (0.48) Change Efficacy: 6.22 (0.69) Personally Beneficial: 6.33 (0.58)	“…. Sometimes [I] don't necessarily think it should be a question of do you want [to talk to a MBH clinician and instead it should] just be like…part of [the process] to talk about [MBH concerns]….it should be…part of their initial diagnosis [visit].” – *Healthcare Professional* [When faced with MBH concerns] I call the doctor, or [a MBH clinician to] talk to them or sometimes the parents will ask to speak to somebody. That's not really our thing. We can kind of notice that there's an issue and we address it with somebody else, but that's not something that we [in our role] typically sit down and discuss with them.” – *Healthcare Professional* “…[Our MBH clinicians] have pretty much stuck with the [a specific sub-population omitted for confidentiality] …but there's a lot of kids that aren't in the [that] population that come here just as much or more…that could benefit from it.” – *Healthcare Professional*	N/A
TEAM 3^a^ (*n* = 4)	Appropriateness: 6.48 (0.56) Management Support: 5.21 (1.26) Change Efficacy: 6.25 (0.96) Personally Beneficial: 6.58 (0.50):	“The integrated care model [is my preference]. I mean I definitely want to share the same records so I can see notes and refresh on what was discussed. But I want them actually in the same work room and same visit so that I can real time have input from their visit [with the patient/family].” –*Administrator* “I know we get a lot of feedback from parents when we go in [to introduce our roles on the medical team] and then [the MBH clinician] introduces herself, they’re very pleased with having that type of team approach. And I think it's been very wellreceived.”–*Healthcare Professional* “…Before there was a [MBH clinician], we dealt with these things the best that we could. And of course we'd refer to outside agencies, but I don't think that the focus was as keen or the awareness was as acute without them..Definitely.” –*Healthcare Professional*	Level 5 Integrated (a particular clinic within Team 3)
			Level 3^b^ Co-Located (all other clinics within Team 3)
TEAM4^c^ (*n* = 2)	Appropriateness: 6.75 (0.07) Management Support: 5.42 (1.06) Change Efficacy: 6.50 (0.71) Personally Beneficial: 7.00 (0.00)	“[The MBH clinician] is usually asked to help with kids who are having psychological contributors that are impacting their medical problems or psychological stuff going on that the team has gotten a sense of and feels like it's not being addressed. That may or may not actually be related to what they have going on medically…the treatment is both psychological and medical and you have to do both…and if you don't do both parts, they don't get better.” – *Healthcare Professional* “…There are times when [the MBH care] transfers over to that co-located model because yes, the [medical and MBH clinician] share an office, yes we see the same patients and [the MBH clinician] also needs time to maybe see a patient for therapy at visits where they're not seeing the [medical team] and see them for a psychological assessment. So, we're physically there together, but we're not necessarily working in that team setting all the time…” – *Healthcare Professional*	N/A
TEAM 5 (*n* = 4)	Appropriateness: 6.38 (0.68) Management Support:5.63 (1.01) Change Efficacy: 6.38 (0.64) Personally Beneficial: 6.67 (0.47)	“[We deal with MBH concerns] on a regular basis. Managing the mental state of the patients and the families in the hospital is always really challenging just because of the nature of the beast…[MBH symptoms] come out, unfortunately toward the nurses, in a lot of negative ways. So that's desperately why I really, really want more psychologists at our hospital or a psychologist for my own [team].” – *Administrator* “We have interns coming in from [MBH to] sit down with [the patients and families].. It's a great outlet for them. But I feel like it helps us be closer with our patients by doing it ourselves also, because then they feel like they're being heard and understood by all members of the team. I feel like it makes us a more close-knit team by [being] able to address these issues [together]. [Our interns are awesome and we sometimes have shared visits]…Talking to two people, they may get more than one point of view…help[s them] understand what's going on, is a positive thing…I really prefer what we do now. Like I said, I feel like it just makes our patients feel more…they trust us more. That bond is stronger. I feel like they feel more at ease talking to us about things because they’re comfortable with us.” – *Healthcare Professional*	Level 4 Co-Located
TEAM 6^a^ (*n* = 3)	Appropriateness: 6.47 (0.21) Management Support: 4.67 (2.60) Change Efficacy: 6.22 (0.25) Personally Beneficial: 6.78 (0.38)	“Integrated care [is my preference]…I would love to have a psychologist integrated right in our team. And he's a person who sees every single patient in our clinic, maybe not every visit, but gets to know families and patients and can then figure out or help them [with]…whatever they need. It's a reliable, trusted, very qualified person who is not going to change. I mean, I’d love to hire someone who could integrate and be willing to do that.” – *Administrator* “I don't think anything [with our integrated approach] needs to change. I think that it's something that we're finally feeling like it's starting to make a difference just because when [the MBH clinician] first started, she had to work. There wasn't a template that she worked from. I think her being involved with outside resources and also with the schools is something that's really positive. I would like just to see it continue to strengthen our relationships with the schools and the different mental health agencies as well.” – *Healthcare Professional*	Level 4^b^ Co-Located (Current)
			Level 5 Integrated (Previous)
TEAM 7 (*n* = 4)	Appropriateness: 5.78 (0.54) Management Support: 4.33 (0.33) Change Efficacy: 5.54 (0.57) Personally Beneficial: 6.58 (0.42)	“Ideally… inpatient integrated care..For outpatient, you can actually have [flexibility with other models, with the caveat of] not just putting an order in and wondering what happens..I guess if you're talking ideals, it should be integrated care as well in the outpatient center, but co-located care is good too.” – *Administrator* “I would like to have someone here and there and.. Because most of our patients…have high risk or difficult issues [medically, developmentally, and psychosocially]. So, even if they don't show things, I would like to have some kind of assessment…and psychological support that is there for the kid and his family. So, I would love to have somebody dedicated for the [specialty name] clinic.” – *Healthcare Professional*	Level 4 Co-Located
TEAM 8^a^ (*n* = 4)	Appropriateness: 6.48 (0.38) Management Support: 4.92 (0.75) Change Efficacy: 6.29 (0.50) Personally Beneficial: 6.00 (0.98)	“I think one [benefit of integrated care] is it instills confidence because unfortunately a lot of these families sometimes have heard a lot of different things from [medical teams]…I think so having that sense of a comprehensive evaluation that we're looking at this from multiple different angles. Being able to explain to the family those things that we're looking at really helps buy-in because I think your first part with families is a buy-in to what you think is going on with child before you can take that second step of what to do about it.” – *Administrator* “I think it would provide a more supportive and comprehensive care. I think it would help them trust what we're doing a little more in that they could see that we really do care about them entirely, not just about one section of their. So, I really do think it would benefit the families tremendously .. I will tell you there are probably going to be patients on the other side though that are so against counseling that they would find this as a reason to not want to seek our care. But if that's the case, they can find [another healthcare professional] or talk to somebody about not receiving that part of care.” – *Healthcare Professional*	Level 6 Integrated (one clinic within team)
			Level 4^b^ Co-Located (all other clinics within team)
TEAM 9 (*n* = 3)	Appropriateness: 6.10 (0.17) Management Support: 4.61 (1.08) Change Efficacy: 5.61 (0.19) Personally Beneficial: 5.89 (0.51)	“Space, money, all those reasons… are the biggest barriers right now. A big part of what's going to drive…[increased access and feasibility of integration of MBH care] is probably…the future of how we do healthcare in this country… As long as we're in the sickness care model, where it's fee for service, when you have symptom X then you seek service Y, that's always going to be [viewed as] more efficient than if you have a wellness model where the goal is to provide whatever care from whatever source will keep the patient in the healthiest state.” – *Administrator* “It would be great to have [a MBH clinician] there all the time, even if it was somebody that's shared amongst a couple of specialties…[there are] a couple patients probably every clinic that could definitely benefit…to [spend some time with them to]identify some of those red flags and…get them to the appropriate place…That's where we would lose momentum. You've got somebody in front of you right now…but then you move onto the next person and if you can't get [the patient] an appointment right away or know the right person to contact, it just falls through the cracks.” – *Healthcare Professional*	Level 4 Co-Located

a Range = 1–7, with higher scores indicating greater readiness for change on a given domain.

b Quotes were reduced for brevity. Context provided in brackets is based information directly pulled from their conversation and used actual language/verbiage, whenever possible.

c Secondary IPAT scores were due to a specialty area having one clinic with a higher level of coordination/integration or a recent change in their services, resulting in a pre- and post-change IPAT scores.

d Secondary IPAT scores were due to a specialty area having one clinic with a higher level of coordination/integration or a recent change in their services, resulting in a pre- and post-change IPAT scores. When considering the overall area of medical specialty, the level of integration observed most consistently across clinics (i.e., Team 1) or the current level after a recent change to team makeup (i.e., Team 6) was used as the primary IPAT score.

e Due to the nature of Teams 2 and 4, an administrator was not required to participate.

Overall, healthcare professionals reported an ardent desire for increased MBH support and collaboration with MBH clinicians in their routine practice regardless of current levels of MBH coordination/integration. Healthcare professionals and administrators described benefits of MBH services across organizational, healthcare professional, and patient/family levels, though recognized important barriers to adoption across the spectrum of care. Consistently, quantitative findings suggested high readiness for change. Qualitatively and quantitatively, teams reported prior experience with MBH services typically at the Co-Located level, as consistent with IPAT scores. Some teams experienced varied access to MBH clinicians over time or across settings, such as varied access based on the day or clinic within a specialty area or changes in funding for this role over time (i.e., a grant-funded position). While only clinic within a team reached the highest level of integrated care, most participants qualitatively described integrated care as the “ideal” model of care.

Findings suggest participants perceived MBH coordination/integration or increased access to these services as worthwhile, with a legitimate rationale. Healthcare professionals described MBH services as appropriate and relevant in the context of pediatric subspecialty medicine and beneficial to the larger organization, specialty team, themselves as individual healthcare professionals, and the patients and families they serve. Many participants, though especially those identifying as MBH clinicians or healthcare professionals with experience with integrated care models, discussed benefits of MBH access such as improvement in medical and psychosocial outcomes among patients and families, the promotion of positive health behaviors (e.g., adherence, self-management), and successful transition to adult care. Participants also highlighted how access to MBH care can decrease risk for worsening MBH symptoms and medical complications, the need for higher-level intervention in the future, and overall reduction in healthcare costs.

High levels of confidence around a team's ability to make a change in the model of MBH care) were also observed quantitatively and qualitatively. Higher RCQ (*Change Efficacy)* scores were consistent with reported interest and willingness to problem-solve and consider practice changes to support increased MBH access. However, administrators often expressed concern that organizational support may hinder these efforts, consistent with *Management Support* scores being the lowest domain for both non-administrative healthcare professionals and administrators. At the same time, some healthcare professionals and administrators also described the value for continuing to advocate for increased access to services to higher levels of hospital administration and recognized some potential concerns of inconsistent access over time (i.e., establishing psychosocial screening procedures and then losing the support to address identified needs sufficiently). A few participants who made these statements described how inclusion of MBH clinicians on teams supports the efficiency of the team, allowing each healthcare professional to act within their scope of practice. These participants noted they are often not sufficiently trained to address MBH concerns (e.g., suicidality, substance use), though these concerns routinely arise in their care of patients and families. Relatedly, significant differences between healthcare professionals and administrators regarding the *Personally Beneficial* subscale of the RCQ were observed in qualitative data, supplementing administrators' qualitative responses of interest and empathy for patients and families about the need for increased access to MBH services and advantages of implementation. This result is clinically significant in that administrators had overall higher levels of experience upon which to base their opinions and perspectives.

Interestingly, when presented with a verbal description and definitions of each care model, healthcare professionals who were not MBH clinicians often had difficulty identifying the current care model of their practice. This confusion resulted in discrepancy between administrator IPAT scores and qualitative reports by healthcare professionals (vs. administrators). For example, it was observed that those on Level 3 Co-Located Care teams and a few healthcare professionals on Level 4 CoLocated Care teams (based on IPAT scores) were often likely to describe their teams as subscribing to a coordinated care model (i.e., Level 2 Coordination, Basic Collaboration at a Distance). Based on further review of these discrepancies, it is also possible that teams had difficulty distinguishing between the two when operating in a capacity that uses multiple models. For example, several teams described different clinic days or times in which MBH clinicians were present and other times in which they were not. On the days without a MBH clinician, teams were more likely to refer patients to community services or internally to the MBH department (more coordinated, focused on written referral-based communication). Conversely, for clinics with an MBH clinician present, communication was more regular and face-to-face, but the MBH clinician held more of a consultative role for the team. Healthcare professionals often described the MBH clinician as an important part of their team, though called upon as needed. Participants also described MBH clinicians as helpful in responding to a team member's thoughts, ideas, or question, in addition to being someone who could address MBH concerns separately while the remainder of the team focused more on medical needs. As these examples demonstrate, while these healthcare professionals often initially identified their teams as having a more coordinated model of MBH care, they often described all key elements of co-located care, consistent with their team's IPAT scores.

Additionally, while integrated care was often described as an “ideal” scenario, it was more frequently identified as unrealistic in participants' setting due to perceived organizational constraints (e.g., time, money, space, number of available MBH clinicians, other resources). This often led to conversations about perceived benefits of lower care models or a preference for colocated care.

## Discussion

The current study examined pediatric specialty healthcare professional and administrator perceptions and experiences with MBH coordination/integration and readiness to change. Extant research on coordinated/integrated MBH is robust, though often focuses on primary care or specific pediatric or adult populations (37–40). Further, while more routine in implementation science and primary care research (41), fewer specialty-medicine-focused studies have used a rigorous mixed methods approach to investigate these factors; included a wide range of healthcare professionals and administrators from various specialty areas, training backgrounds, and experiences with MBH services; and targeted a rural, Appalachia-serving hospital system. Despite this, several qualitative and quantitative studies, trials, systematic reviews, and metaanalyses have explored the benefits of and barriers and facilitators to implementing MBH services into medical contexts (40). Findings suggest the inclusion of MBH support on medical teams improves patient outcomes and reduces healthcare costs (37, 42, 43). Research has also demonstrated how strategies, such as cost-effectiveness analyses, can be used by pediatric psychologists and other MBH clinicians to demonstrate the beneficial economic impacts of their work (44). Still, widespread integration MBH services in pediatric subspecialty medicine is far from reality for many healthcare settings, particularly in rural settings where patients are more likely to experience mental health concerns, less likely to receive treatment, and experience higher rates of suicide-related deaths (45).

While an investment in expansion of MBH services has demonstrated reduced overall healthcare costs and preventive impacts on healthcare utilization (46), participants in this study also emphasized finances as a key barrier to the adoption, implementation, and sustainability of MBH integration. Increased awareness of the benefits of integrated care on a financial level, particularly in rural settings like Appalachia, appears to be an area of future need particularly as comments such as little to no reimbursement for MBH visits were noted by several healthcare professionals/administrators. While some specialties/teams developed unique strategies to circumvent financial barriers (i.e., soliciting external grant funding, using trainees from local graduate programs as MBH clinicians), these approaches pose sustainability barriers. This was highlighted in both qualitative and quantitative data obtained from Team 6, whose level of coordination/integration changed following the loss of an external MBH grant. As such, it is likely that conversations, collaboration, and advocacy at the organizational, payor, and policy level are warranted, detailing the different options and approaches to coordination/integration care possible as well as the best strategies for both implementation and sustainability. Further, given known disparities in MBH access, particularly for rural, Appalachian contexts, facilitating incorporation of MBH access in other, ongoing health equity efforts is paramount.

During analysis, we also observed some confusion around coordinated/integrated care terminology, particularly for non-administrative and non-psychologist healthcare professionals. When provided with a copy of each definition, participants had minimal to no difficulties identifying or describing key components of integrated care, and participants provided specific examples of these care levels in their or others' practices. Similarly, no major difficulties were observed for the other side of the continuum with understanding the lowest levels of coordinated care. Healthcare professionals who perceived their clinic or specialty area to be integrated in nature also correctly identified their teams as such when compared to team IPAT scores.

Conversely, non-administrative healthcare professionals in fields other than psychology had more difficulty distinguishing between the higher level of coordinated care and the lower level of co-located care. Comments suggest this discrepancy may be related to the shared emphasis on basic collaboration and differing conceptualizations in specialty medicine (vs. primary care) of “separate facilities” and “onsite” given the context of a hospital system divided by departments and divisions, and variation in office spaces, locations, and clinic layouts. This confusion often resulted in participant perceptions that MBH services were less coordinated/integrated (at a lower level) than objectively calculated IPAT scores.

These findings are consistent with other previous work which has demonstrated that healthcare professionals and patients alike are confused by terminology and the experience of integrated care models, but are clear on the overarching concept and its active components or impacts on the team as a whole (47, 48). Findings are also consistent with work on co-located and integrated care models, suggesting the main predictor of positive perceptions of successful interprofessional collaboration in healthcare contexts is co-location of MBH care (49). This is often why formal trainings, especially in primary care contexts, have been developed to teach healthcare professionals about coordinated/integrated MBH models (47). Similar trainings may prove beneficial in pediatric specialty environments prior to adoption/implementation or changes to current MBH coordination/integration.

The observed differences (lower levels) of *Management Support* in the study as compared to other RCQ domains is also an important finding. Healthcare professionals often reported beliefs that their primary manager/administrator was supportive of MBH collaboration and organizational barriers were perceived to be greater at higher levels of hospital administration. Consistently, Administrators often expressed great enthusiasm and interest in increasing MBH supports within and outside of their specialty areas, though readily detailed organizational barriers at the hospital and higher policy/public health system levels (e.g., structure of the hospital system itself, U.S. healthcare structure/payment systems). Future work could benefit from including more senior managers and healthcare leaders when investigating barriers and facilitators to MBH integration. Research using focus groups or other shared learning methodologies, in addition to continued mixed methods designs may also improve our understanding of readiness to change regarding MBH integration across and within levels of a healthcare system/organization.

### Limitations

The present study is not without its limitations. First, as the design was exploratory and predominately emphasized the qualitative components of the mixed methods design (QUAL-quant), the sample size limited the statistical plan and quantitative hypothesis testing and thus the level of comparisons included in our mixed methods analysis. Relatedly, given the number of MBH clinicians eligible to participate at the time of this work, our team was unable to parse out unique perspectives of MBH clinicians by type (i.e., psychologists vs. other healthcare professionals) in this manuscript without impacting participant confidentiality. Further, though several attempts were made to decrease conflicts of interest and social desirability (i.e., assigning interviewers with no professional working relationship with the participant), some healthcare professionals had some interactions with other authors/study investigators outside of the study. Further, as described for transparency in the author note, qualitative analyses may have been impacted by investigator worldviews, backgrounds, and experiences. Lastly, this study focused on a single healthcare system predominately serving rural Appalachia. These sociodemographic considerations are important, as they potentially impact applicability of findings to other settings or healthcare systems. However, applicability and generalizability are not the primary objective of qualitative or mixed methods research.

### Strengths and contributions to mixed methods research, clinical practice, and policy

This study also has several strengths and contributions. First, an evidence-based approach was taken to study design, data collection, and data analysis. Validated measures were used to assess most constructs and non-validated tools (e.g., semi-structured interview guide) were piloted and modified based on feedback from individuals with relevant expertise. Purposive and theoretical sampling techniques were used for codebook stability and diverse representation of participant backgrounds. Real-time data also informed sampling decisions. These features are innovative for the integrated MBH care literature, where emphasis has been placed on primary care/a single pediatric subspecialty area; observational methods, qualitative, or survey data alone; and non-validated, study-specific tools (40, 50, 51).

The rigorous, empirically based convergent mixed methods design of this study, however, resulted in rich, comprehensive data and the ability to identify consistencies and contradictions across qualitative and quantitative reports. Taking a QUAL-quant approach also ensured findings were grounded in the experiences and voices of participants. Finally, the focus on a healthcare system in a historically marginalized and underserved community (i.e., rural Appalachia) allowed for unique examination of the construct (MBH coordination/integration) and its variation when a healthcare system has limited access to resources. Other studies often emphasize larger, well-resourced healthcare systems with more widespread adoption or structures in place to support higher MBH coordination/integration.

Across psychology, medicine, and mixed methods literatures, there is growing recognition of the strengths, applicability, and practical utility of mixed methods research to dissemination/implementation and health equity science, especially as it relates to reducing MBH science-to-practice gaps (52, 53). This manuscript applied mixed methods to a quality improvement initiative aimed to understand multilevel factors influencing MBH coordination/integration in pediatric subspecialty care. Consequently, our project resulted in data aligned with several of Dahler-Larsen's (54) proposed practical utilities of mixed methods research. Specifically, the mixed methods approach applied in the present study: (1) provided nuanced understanding of constructs and new insights; (2) produced the type of practical knowledge that best serves organizational partners, such as healthcare professionals and leadership; and (3) identified and catered to the diverse perspectives and needs across employee/manager (i.e., Healthcare Professional/administrator) types, including expression of joint commitment and conflicting views (54).

As previously stated, the present study was also designed with quality improvement in mind, and the potential to have impact on the healthcare system from which we sampled. Prior to this publication, results were shared with study participants, administrative leaders, and the larger pediatric faculty to spur ongoing conversation around MBH support within the system and inform recruitment efforts for additional MBH clinicians. Moreover, the present study serves as an exemplar for how to conduct rigorous, yet low-cost MBH-related research within a hospital system.

Overall, data suggests partnerships between healthcare professionals, leadership, and policymakers is warranted to address barriers and improve MBH access and equity in these settings, including improving policies related to fiscal resources (i.e., funding, reimbursement), productivity metrics/time (e.g., billable hours and relative value units); education and training related to MB; and protecting time to identify preferred models of MBH care, learn to collaborate with MBH clinicians, assess implementation outcomes, and refine procedures over time to support sustainability. Moreover, data facilitated understanding of the multilevel factors influencing MBH implementation and provided guidance for future research, clinical, and policy directions aimed to reduce MBH disparities.

## Conclusion

Chronic illness and medical complexity places unique demands on patients, families, and the medical system; often coincides with increased risk for psychosocial concerns overall; and contributes to healthcare disparities. Future research should explore barriers and facilitators to MBH integration longitudinally and with various partners beyond healthcare professionals to include higher-level administrators/hospital leaders, policymakers, and patients/families. Specialty medicine conducted in rural and other marginalized contexts presents unique challenges and strengths which should also be explored further as should how lack of access to MBH exacerbates MBH concerns and places an undue burden on healthcare professionals without MBH training to address these needs as part of their care despite scope of practice, experience, and comfort with doing so. Future research could also involve comprehensive needs assessments or cost-benefit analyses to quantify gaps between current and desired MBH landscape within healthcare organizations; obtain additional context around systemic/logistical barriers and facilitators (e.g., cost, resources, time, space); and understand the feasibility and sustainability of different models of MBH coordination/integration.

In the present study and beyond, de-identification and limiting participant demographics are common strategies used to protect participant confidentiality. However, future research should consider novel methodological approaches to explore within- and between-group differences in perceptions while maintaining participant confidentiality, particularly in rural or smaller healthcare contexts. Practical guides and handbooks such as those on Rural Healthcare Ethics (55) can support researchers in thinking through these concerns while balancing benefits and risks. Researchers may also benefit from conducing multi-site studies and incorporating community-engaged approaches (56) by embedding healthcare professionals, administrators, and other key stakeholders into the research team and working together to develop new strategies to overcome and address barriers to confidentiality and support larger, engaged samples in this type of research (57).

## Author's note

In the spirit of self-reflexivity, authors recognize their positionality as highly-educated individuals with employment and/or training in academic (university and medical) environments. Authors also self-identify as a multi-racial, Afro-Latina and Indigenous, cisgender female (DNW); and white, cisgender females (JK, DMD, CLD). We acknowledge the potential influences of these identities on the study design, conduct, and interpretation of findings and implications described in the present manuscript.

## Data Availability

The datasets for this article are not publicly available due to concerns regarding participant/patient anonymity. Requests to access the datasets should be directed to the corresponding author.
